# Case report: Unruptured small middle cerebral artery aneurysm with perianeurysmal edema

**DOI:** 10.3389/fsurg.2023.1134231

**Published:** 2023-04-11

**Authors:** Yoshihiro Goto, Yoichi Morofuji, Eri Shiozaki, Daiki Uchida, Ichiro Kawahara, Tomonori Ono, Wataru Haraguchi, Keisuke Tsutsumi

**Affiliations:** Department Neurosurgery, National Nagasaki Medical Center, Omura, Japan

**Keywords:** aneurysm rupture prediction, clipping, perianeurysmal edema, fluid-attenuated inversion recovery, middle cerebral artery (MCA)

## Abstract

**Background:**

Perianeurysmal edema (PAE) has a tendency to occur in embolized aneurysms but also in partially thrombosed, large, or giant aneurysms. However, there are only a few cases recorded in which PAE was detected in untreated or small aneurysms. We suspected that PAE might be an impending sign of aneurysm rupture in these cases. Herein, we presented a unique case of PAE that was related to an unruptured small middle cerebral artery aneurysm.

**Case description:**

A 61-year-old woman was referred to our institute due to a newly formed abnormal fluid-attenuated inversion recovery (FLAIR) hyperintense lesion in the right medial temporal cortex. Upon admission, the patient did not present with any symptoms or complaints; however, FLAIR and CT angiography (CTA) suggested an increased risk of aneurysm rupture. Aneurysm clipping was conducted, and no evidence of subarachnoid hemorrhage and hemosiderin deposits around the aneurysm and brain parenchyma was noted. The patient was discharged home without any neurological symptoms. MRI taken at eight months post-clipping revealed complete regression of the FLAIR hyperintense lesion around the aneurysm.

**Conclusion:**

PAE in unruptured, small aneurysm is thought to be an impending sign of aneurysm rupture. Early surgical intervention is critical even for small aneurysms with PAE.

## Symptoms

Asymptomatic; physical exams: normal; lab results: aneurysm growth and bleb formation, fluid-attenuated inversion recovery (FLAIR) hyperintense lesion around the aneurysm.

## 1. Introduction

Subarachnoid hemorrhage (SAH) following an intracranial aneurysm rupture results in high morbidity and mortality. Although many factors may affect the possibility of a rupture, it remains impossible to predict the timing of aneurysmal rupture. Current evidence has demonstrated that perianeurysmal edema (PAE) has a tendency to occur in embolized aneurysms but also in partially thrombosed, large, or giant aneurysms. The etiology of PAE is poorly understood, but it may be due to a minor leakage, aneurysmal thrombosis, or inflammation in an aneurysmal wall. Some reports have proposed PAE as a precursor of aneurysmal rupture.

We herein present a unique case of PAE, which is related to unruptured small middle cerebral artery aneurysm, and discuss the underlying mechanisms and clinical implications in comparison to previous cases. To the best of our knowledge, this is the first case that successfully prevented the rupture of a small aneurysm due to PAE.

## 2. Case description

**Figure 1:**
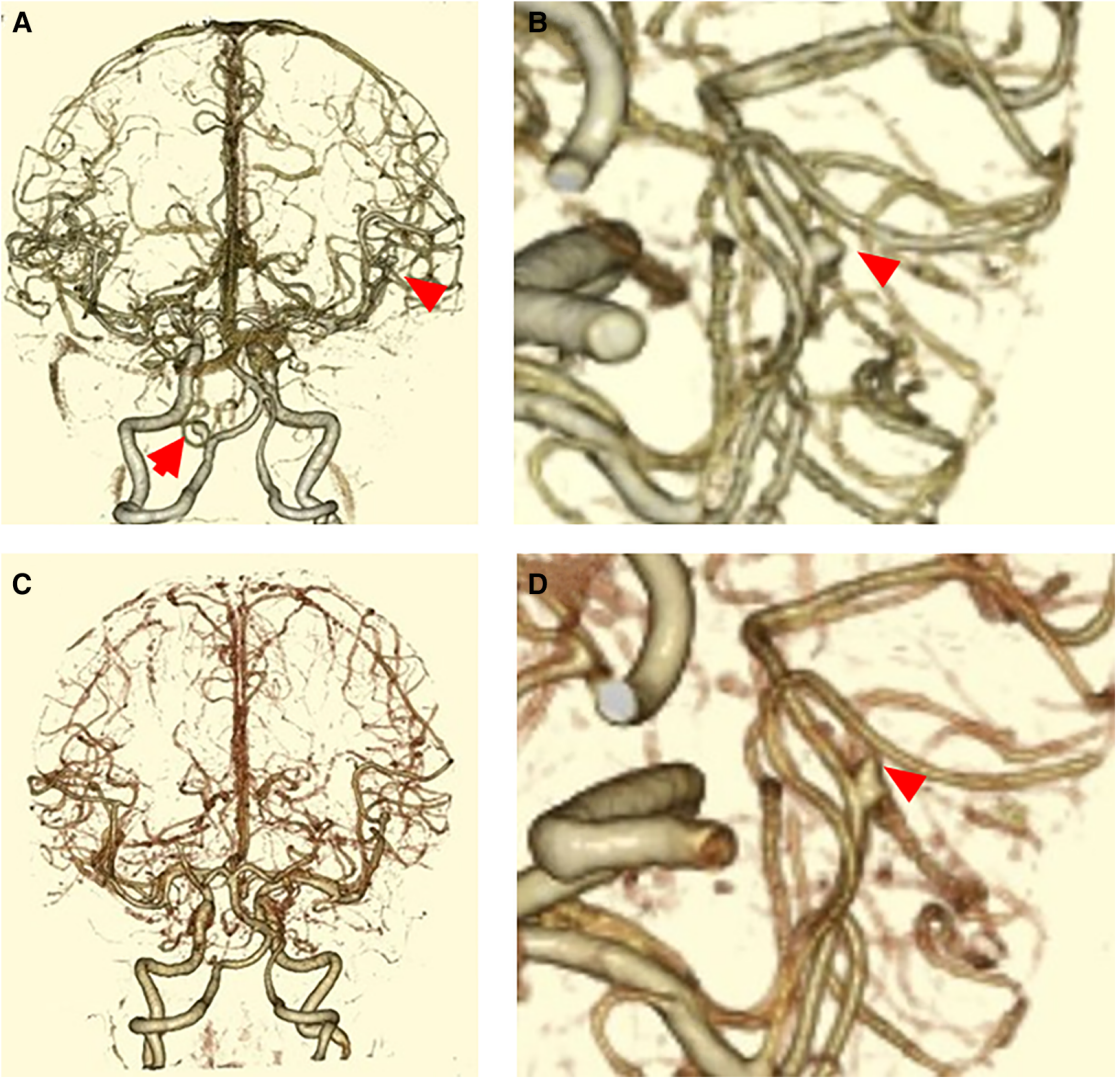
(**A**) CTA obtained two years before this admission (posterior- anterior view). A left VA-PICA aneurysm and a right MCA aneurysm (arrowhead) were noted. The left VA-PICA aneurysm (arrow) was clipped at first admission. (**B**) The scaled-up version of CTA taken two years before this admission. The size of the right MCA aneurysm (arrowhead) was 3 mm. (**C**) CTA obtained at this admission. No *de novo* aneurysm was found. (**D**) The scaled-up version of CTA obtained at this admission. The aneurysm size was 4 mm, and bleb formation was observed (arrowhead).

**Figure 2:**
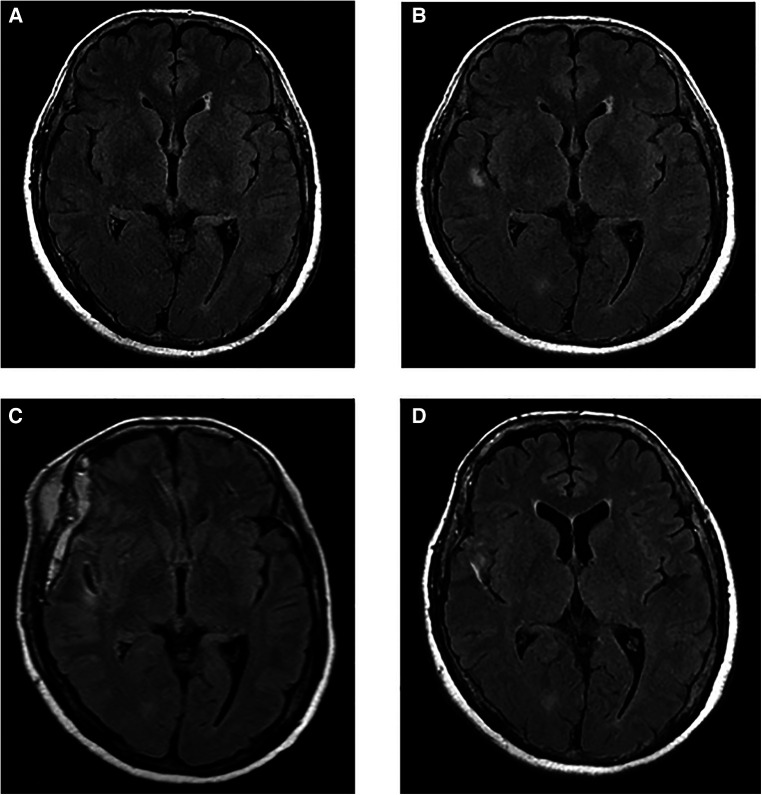
(**A**) MRI-FLAIR image taken six months before this admission. (**B**) MRI-FLAIR image taken at this admission. A FLAIR hyperintense lesion was detected in the inner temporal lobe. (**C**) MRI-FLAIR image taken at post-operative day 7. The FLAIR hyperintense lesion persisted. (**D**) MRI-FLAIR image taken eight months after discharge. The FLAIR hyperintense lesion disappeared.

A 61-year-old female patient with hypertension and hyperlipidemia was referred to our institute due to a newly formed abnormal FLAIR hyperintense lesion in the right medial temporal cortex. The patient had a history of subarachnoid hemorrhage caused by a ruptured left vertebral artery-posterior inferior cerebellar artery (VA-PICA) aneurysm two years prior to admission (Figure 1A). She underwent aneurysm clipping and was then discharged home after one month of rehabilitation with a modified Rankin Scale (mRS) of 0. A 3-mm aneurysm of her right middle cerebral artery (MCA) had been detected (Figure 1B), and the patients had been followed up by MRI every six months at a local hospital. There was no FLAIR hyperintense lesion adjacent to the MCA aneurysm at six months prior to admission (Figure 2A). Upon admission, her clinical and neurological examinations were normal, and she had no symptoms of headache, nausea, or vomiting that would indicate rupture of the aneurysm. Her Glasgow Coma Scale was E4V5M6 and her vital signs were stable. Brain CT and MRI revealed no evidence of subarachnoid hemorrhage. However, brain MRI (Figure 2B) revealed the presence of a FLAIR hyperintense lesion adjacent to the aneurysm, while CT angiography (Figures 1C,D) showed aneurysm growth with bleb formation at the posterior site.

**Figure 3:**
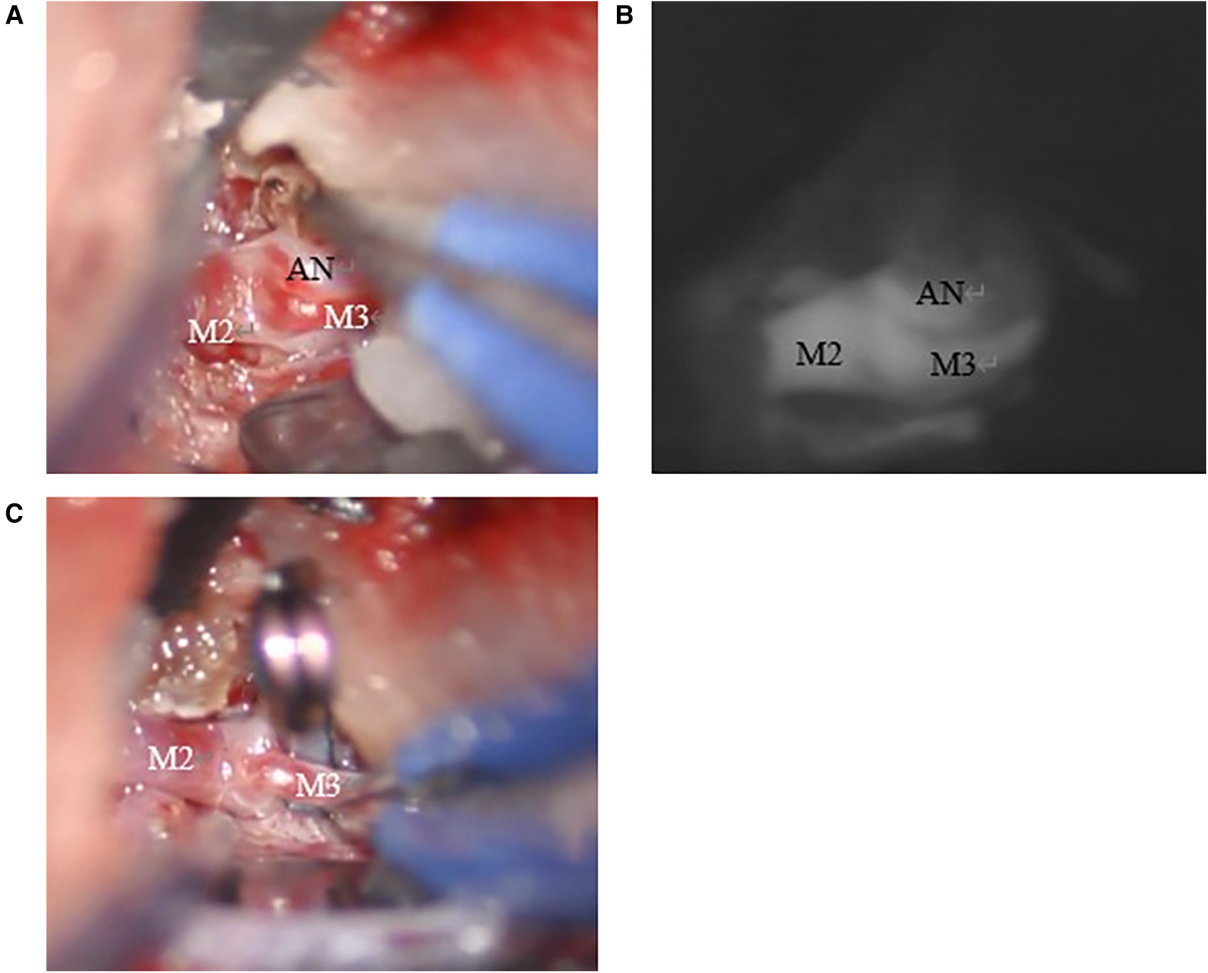
(**A**) The aneurysm was detected on the right M2/3 bifurcation. There were no hemosiderin deposits around the aneurysm and brain parenchyma. (**B**) The aneurysm enhanced with indocyanine green. (**C**) The aneurysm was clipped with Sugita mini side angle blade 6 mm no.104.

These images suggested a high risk of aneurysm rupture. We conducted mild sedation and the patient underwent aneurysm clipping on the day after admission. During the procedure, there was no evidence of subarachnoid hemorrhage, hemosiderin deposits around the aneurysm, and brain parenchyma (Figures 3A–C). MRI scans (Figure 2C) on post-operative day 7 revealed that the FLAIR hyperintense lesion had almost disappeared. The patient was then discharged home without any neurological symptoms on post-operative day 11. MRI scans (Figure 2D) taken eight months after clipping demonstrated complete regression of the FLAIR hyperintense lesion around the aneurysm.

## 3. Discussion

In this study, we identified a FLAIR hyperintense lesion adjacent to distal MCA aneurysm as PAE. There have only been five cases of PAE caused by untreated aneurysms, which resulted in aneurysm rupture (Table 1). Previous case reports showed that PAE indicates the presence of a minor leakage and subsequent rupture, thus early intervention is important (1). Based on these reports, early aneurysm clipping was performed successfully. Although there was no evidence of subarachnoid hemorrhage, hemosiderin deposits around the aneurysm, and brain parenchyma during the surgery, we believe that early clipping of aneurysms with PAE could efficiently prevent aneurysm rupture.

**Table 1:** Six cases of untreated aneurysms with perianeurysmal edema **.**

Case	Age/sex	Presentation	CT at the admission	AN locations and size	Rupture	Treatment	Hemosiderins	Prognosis
Hiu, et al. 2009	71/F	Aneurysm enlarge in 3 years	Pure ICH	BA	+	Coiling	NA	NA
				5 mm				
Pahl,et al. 2014 (case 1)	37/F	Thunderclap headache	SAH, ICH in R-frontal lobe	Acom	+	Clipping	NA	GOS5
				5 mm				
Pahl,et al. 2014 (case 2)	45/F	Sudden thunderclap headache	SAH, ICH in L-frontal lobe	L-ICA	+	Clipping	NA	GOS4
				16 mm				
Asano et al. 2020	79/F	Headache	-	R-MCA	Minor leak suspicious	Clipping	+	mRS0
				10 mm				
Suzuki et al. 2021^a^	65/F	ACA aneurysm rupture	SAH, ICH in frontal lobe	L-MCA	+	Clipping	+	mRS5
Present case	61/F	Subclinical	-	R-MCA	-	Clipping	-	mRS0
				3 mm				

AN, aneurysm; F, female; PAE, perianeurysmal edema; ACA, anterior cerebral artery; ICH, intracranial hemorrhage; SAH, subarachnoid hemorrhage; R-frontal, right-frontal; L-frontal, left-frontal; BA, basilar artery; Acom, anterior communicating artery; L-ICA, left-internal carotid artery; R-MCA, right middle cerebral artery; L-MCA, left-middle cerebral artery; NA, not available in the original publication; GOS, Glasgow Outcome Scale; mRS, modified Rankin Scale.

^a^ This case was determined as PAE with cerebral parenchyma low density area around the unruptured aneurysm by CT and CTA.

PAE is a rare MRI finding that it is generally found in cases of embolized, giant intracranial, and partially thrombosed aneurysms (2–5). Su et al*.* analyzed 124 cases of embolized unruptured aneurysms, and the incidence of PAE was 6.8% (5). Dang et al*.* also analyzed 69 cases of giant intracranial aneurysms (>25 mm) of which 33.3% developed PAE (3). In contrast, there have only been five cases of PAE in unruptured and/or untreated aneurysms (1, 6–8). Among them, four cases experienced aneurysm rupture immediately after the manifestation of PAE, whereas in one case PAE was speculated due to a minor leakage. Hemosiderin deposits around the aneurysm and brain parenchyma were detected in two of these five patients. Among these untreated aneurysm cases with PAE, our case is unique with regards to its small size (3 mm, the smallest among these five cases), absence of SAH and/or hemosiderin deposits, and the subsequent clinical course of the patient, since PAE was completely resolved within eight months postoperatively.

Although the etiology of PAE is still poorly understood, aneurysm wall remodeling is believed to be the main cause of PAE (9). This remodeling leads to repetitive bleeding and thromboses inside the aneurysms, yielding thrombin and hemoglobin degradation products (10), which subsequently infiltrate brain parenchyma and cause edema (11). Other factors involved in the development of PAE are bleb formation, aneurysm enlargement, and inflammatory processes on the aneurysm wall (2, 4, 9). Aneurysm wall remodeling induces a wall inflammatory reaction and brings pressure on brain parenchyma. Compression of brain parenchyma may result in the development of a brain parenchyma edema, which can be detected as a FLAIR hyperintense lesion. At the same time, minor leakage can occur during the remodeling process, and hemosiderin deposits may also induce edema accordingly. Hemosiderin deposits can be considered an indirect sign of minor leakage, which can be a predictive sign of impending aneurysm rupture (1). Contrary to our case study, previous reported cases have observed hemosiderin deposits around the aneurysm and brain parenchyma during the operation (1, 6). Lack of hemosiderin deposits is a crucial finding of our case compared with previous cases because it clearly indicates that PAE can occur without any minor leakage. The absence of hemosiderin deposits around the aneurysm and thrombosis inside the aneurysms suggests that PAE in our case may be associated with bleb formation and mass effect on brain parenchyma. The clinical course and treatment outcomes of our case also support this hypothesis. Previous cases reported that PAE resolved in approximately one-week post-intervention. In contrast, a FLAIR hyperintense lesion could be detected in our case on post-operative day 7, indicating the earlier detection of PAE in our case compared with the previous ones. Incidental earlier detection of PAE resulted in good clinical outcomes. Although we are not sure about exact time that PAE was resolved, we do know that the FLAIR hyperintense lesion was resolved completely within eight months. Considering that Hiu et al*.* continued to observe PAE until aneurysm rupture, our case supports that early surgical intervention is critical even for small aneurysms with PAE.

## Conclusion

The etiology of PAE in our case is unclear, but it is thought to be mainly due to bleb formation and subsequent brain parenchyma compression—and not due to minor leakage. Although we cannot provide straightforward evidence to support this hypothesis, we can suggest that early surgical intervention should be recommended for unruptured aneurysms with PAE to prevent aneurysm rupture, even when the size of the aneurysm is relatively small.

## Data Availability

The original contributions presented in the study are included in the article/Supplementary Material, further inquiries can be directed to the corresponding author/s.
